# Initiation of Absconding-Swarm Emigration in the Social Wasp *Polybia occidentalis*

**DOI:** 10.1673/031.009.1101

**Published:** 2009-04-06

**Authors:** Peter J. Sonnentag, Robert L. Jeanne

**Affiliations:** Department of Entomology, University of Wisconsin, Madison, Wl 53706

**Keywords:** Behavior, Costa Rica, tropical wasp, dispersal, house-hunting, amplification, contagion

## Abstract

When a colony of the swarm-founding social wasp *Polybia occidentals* loses its nest to severe weather or predation, the adult population evacuates and temporarily clusters on nearby foliage. Most of the adults remain inactive in the cluster, while foragers bring in nectar and scout wasps search the surrounding area for a new nesting site. After several hours, the scouts stimulate the rest of the swarm to leave the cluster and follow their pheromone trail to the chosen site. How scouts communicate to their swarm-mates that a site has been chosen and how they induce the swarm to depart are unknown. Video records of six Costa Rican swarms were used to quantitatively document changes in the frequencies of social behaviors leading to swarm departure. This was accomplished by going backward through the video record and following the behavior of individuals prior to their departure. Analysis of the behavior of scouts and inactive wasps indicated an increase in the frequency with which scouts bump into inactive wasps prior to swarm departure, as well as a shift in the behavior of inactive wasps from primarily receiving bumps to bumping others before departure. Thus, bumping is propagated by recently activated individuals before they take off. These observations suggest that not only is bumping an activation stimulus that causes swarm members to depart for the new nest site, but it is contagious, leading to its amplification throughout the swarm.

## Introduction

In social insects that initiate colonies by means of swarming, three organizational challenges must be faced when swarms disperse. The first is that a subset of the population, the scouts, must find and agree upon a suitable nest site. Second, the scouts must induce the rest of the colony to leave for the new site. Finally, scouts must guide the migrating swarm of adults to the new site. The process has been best studied in the honey bee, *Apis mellifera*. Scout bees leave the temporary cluster to search for potential nesting sites. After several hours or days, a single site is agreed upon based on quorum sensing by scouts; that is, scouts favor a site at which they observe a sufficient number of other scouts visiting (Seeley and Visscher 2004). Once a site is selected, scouts return to the swarm and prepare it for liftoff by rapidly running over the clustered bees and piping (producing short, rising sound pulses) (Seeley and Tautz 2001). The actual liftoff of the swarm, however, is triggered by buzz running, wherein excited bees run in zigzag patterns while pushing their way through the cluster and occasionally buzzing the wings (Lindauer 1955; Esch 1967; Seeley and Tautz 2001). Within a few minutes, the entire swarm takes to the air and is then guided by scouts, which streak through the flying swarm in the direction of the new nest site (Beekman et al. 2005).

Much less is known about the emigration process in swarm-founding social wasps. Best studied in this regard is the Neotropical species *Polybia occidentalis*. In Guanacaste, Costa Rica, reproductive swarms typically depart from the natal nest at the end of the wet season (Forsyth 1978; Jeanne 1999). Absconding swarms, however, may be produced at any time as a result of brood-raiding by ants (Bouwma et al. 2007) or when a nest is damaged beyond repair by a vertebrate predator, severe weather, or accident. The entire adult population abandons the nest, at first scattering widely, but within 30 minutes coalescing into a single dense cluster on a leafy twig near the nest (Bouwma et al. 2000, 2003). Scouts then begin to explore the surrounding area for a suitable nesting site (Bouwma et al. 2003). Scouts appear to be drawn from the ranks of foragers (Forsyth 1981; West-Eberhard 1982). They inspect several sites at first, but within a day or so narrow the choices to one. After the scouts have reinforced their trail to the chosen nest site by leaving pheromone spots on leaves, they induce the other wasps in the cluster to follow the trail (Jeanne 1975; Naumann 1975). Unlike in honey bees, the clustered individuals of *P. occidentalis* leave their resting site one by one over a period of 10 to 30 minutes and follow the trail individually (Jeanne 1981a; PJS, personal observation). If there are males in the swarm, they remain behind (Bouwma et al. 2000). Upon arrival at the new site, workers immediately begin construction of the new nest (Jeanne and Bouwma 2002).

Still unknown is how the scouts communicate to the cluster that a site has been chosen and how they stimulate inactives to leave for the new site. Here, for *Polybia occidentals,* we describe the behavior of wasps on the swarm cluster during the period leading up to and including liftoff. The mean frequency with which scouts bump others in the swarm was found to increase as the departure approaches and that wasps in the cluster shift their behavior from inactivity and receiving bumps to moving over the cluster and bumping others before taking off. This is the first detailed, quantitative study of preemigration swarm behavior for a social wasp and the first systematic study done on multiple swarms of the same species.

## Methods

### Location

The study was conducted at Las Pumas, a private estate 5 km west of Cañas, Guanacaste, Costa Rica (10°25′N, 85°7′W). Cañas is located in the tropical dry forest life zone and has a wet season extending from May into November. Much of the native vegetation at the site was replaced by pasture, but tropical dry-forest species were still represented. The colonies used in this study nested in trees and shrubs.

### Swarming and videotaping

The study site consisted of a pasture containing a scattering of low trees, predominantly *Guazuma ulmifolia* (Sterculiaceae). Active nests of *P. occidentalis* were moved into this area for ease of observation. The twig bearing the nest was cut and carried to the study site, where it was spring-clipped to a low twig in a tree in the pasture. Each translocation was done after dark to ensure that all adult wasps remained inside the nest.

**Figure 1.**
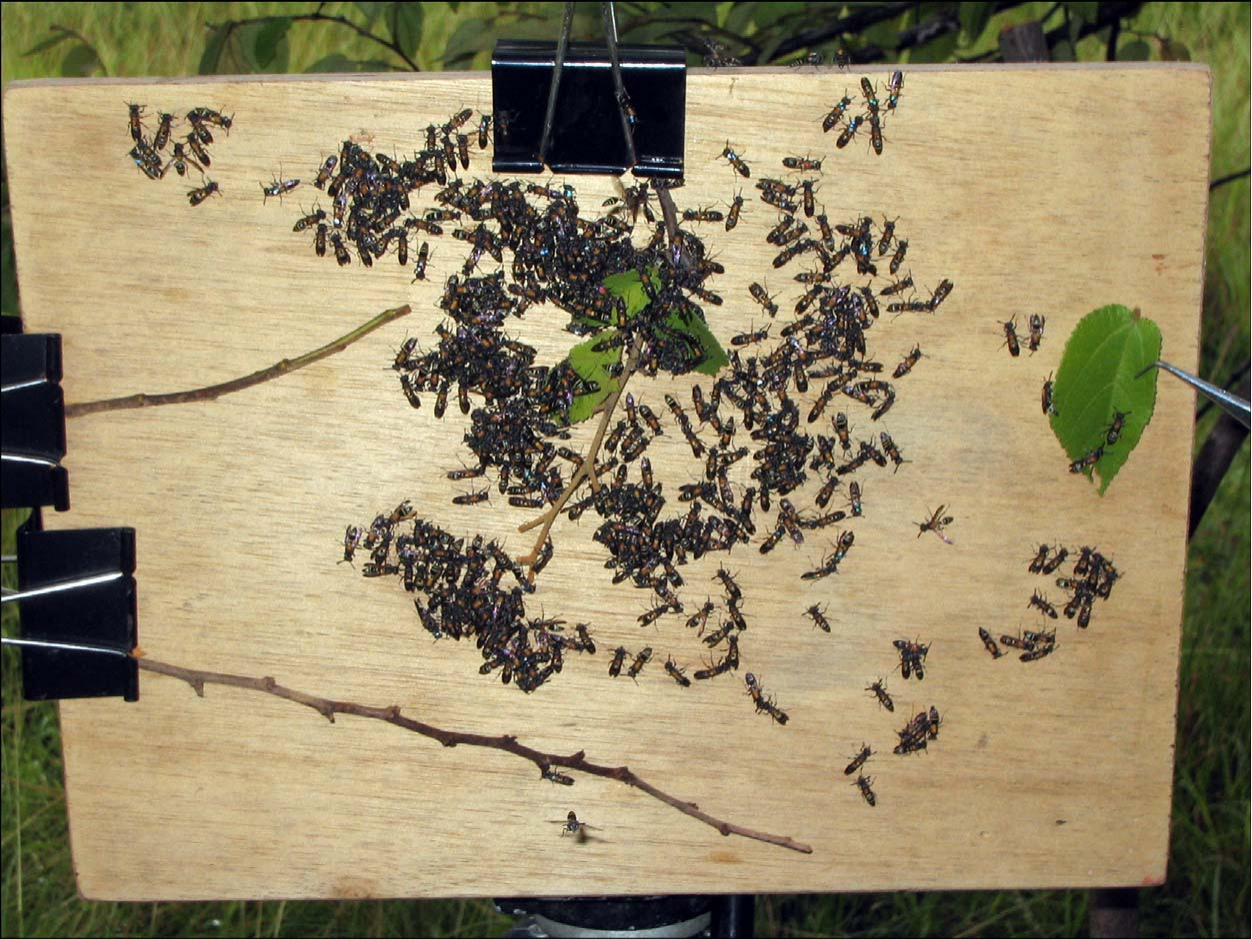
The swarm-observation board. Branch bearing the swarm has been spring-clipped to the board and leaves and twigs are being carefully removed one by one to coax wasps onto the flat surface for video recording of behavior.

The day following the move, the colony was induced to swarm by dismantling the nest in mid-afternoon. This gave the absconding population time to coalesce into a single cluster, but not enough time to find and move to a new nest site before dark. The size of the swarm was determined using a highly accurate predawn census technique described elsewhere (Bouwma et al. 2000). After the swarm had re-clustered following the census, its supporting twig was cut and spring-clipped to a wooden board (30 cm wide × 22.5 cm high) mounted vertically on a tripod and placed in the shade of the tree near where the swarm had re-clustered. We coaxed the swarm from its twig and onto the board by carefully removing one leaf at a time (Figure 1). With the removal of the last bit of twig, the swarm formed a cluster no more than two wasps deep on the board. At this point we began videotaping the swarm, using a digital camcorder (Sony DCH-HC1000) zoomed so that the board almost filled the LCD screen of the camcorder. Videotaping continued until the last wasp departed from the board. Behavioral data were taken by viewing the tapes on a digital videocassette recorder (Sony DHR-1000). One swarm was studied in 1998 and five in 2005. An additional swarm on a twig was videotaped and the video reviewed to determine whether the behavior of unmanipulated swarms differed qualitatively from that of our manipulated swarms.

Preliminary study of the videotapes allowed the discrimination of three categories of individuals on the swarms. “Scouts” were defined as individuals that landed on the board and spent time ranging from a few seconds to several minutes walking or running over the clustered wasps, bumping into them, and stopping only occasionally and briefly to engage in trophallaxis before taking off again. “Foragers” landed on the board and moved more slowly over the clusters of inactives for up to several minutes at a time. They stopped frequently to engage in trophallaxis with both inactives and scouts, but did not exhibit bumping behavior. “Inactives” comprised the largely unmoving wasps clustered densely on the board. These constituted the majority of each swarm and presumably included queens, males, and younger workers. Despite their designation, inactives did in fact become active shortly before departing, as we describe below.

The total number of wasps present on the board was determined every ten minutes by pausing the digital tape of the swarm and counting. This record was used as a measure of emigration progress against which scout behavior was compared. The beginning of emigration was defined as the start of the 10-minute sample period in which the number of wasps present on the board showed at least a 15% drop from the previous 10-min period.

All variables were plotted against time to reveal changes in behavior leading up to and through departure of the last wasp from the board.

### Behavior of scouts

**Figure 2.**
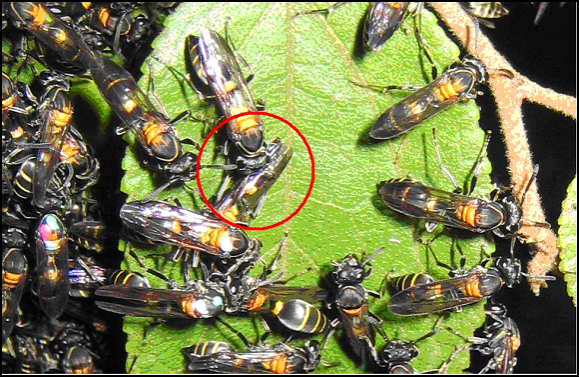
Bumping behavior by *Polybia occidentalis*. The upper individual in the circle is forcibly running into the gaster of an inactive wasp in an absconding swarm. Some wasps are marked with paint spots.

In each swarm five scouts were sampled for detailed behavioral analysis every 20 minutes throughout the preemigration and emigration periods recorded on videotape. The scouts selected were the first to land in each of the first five consecutive minutes of each 20-minute period. For each scout the number of bumps it issued during its entire stay on the swarm was recorded. A bump was defined as the butting of the head against any part of the body of another individual in the cluster (Figure 2). No attempt was made to discriminate “intentional” bumps from “incidental” bumps. From these data, bumping frequency (bumps per minute) was calculated for each scout and the mean computed for each set of five scouts. In addition to sampling individual scout behavior, overall scout activity level was quantified as the number of scouts landing on the board during the first five minutes of each 20-min interval.

### Behavior of inactives

To explore what might stimulate an inactive wasp to become active and depart, the pre-takeoff behavior of a sample of inactives was analyzed from each swarm. Wasps were selected for the sample by playing the videotape in reverse from the end of emigration and identifying individuals that were not only seen departing from the swarm, but remained clearly visible on the video screen for a full five minutes before departure. Fifty individuals were sampled in each swarm, taking care that they were distributed as uniformly as possible across the entire emigration period. Each selected individual was followed backwards on the tape for five minutes prior to its takeoff. The tape was then played forwards while scoring the behavior of the focal individual at each second up to takeoff. Scored behaviors fell into one of the following categories: inactive (unmoving), moving (walking on the board or cluster), bump received (bumped by another individual), bump given (focal individual bumps another individual), or trophallaxis (mouthpart-to-mouthpart contact with another wasp). Inactives were reliably distinguished from scouts and foragers by being present on the cluster and inactive five minutes before departure. Individuals identified as scouts or foragers were omitted, leaving 35–43 inactives in each sample.

For each swarm, the occurrences of each behavior were summed for all individuals for each one-second interval. These sums were then binned into five-second intervals for smoothing. The mean number of inactives performing each behavior in each five-second interval was calculated. Results are plotted against time as the proportion of inactives performing a given behavior (mean number of inactives performing the behavior divided by the number of inactives in the sample). This can be interpreted as the probability of an inactive's engaging in that behavior in that time interval.

In addition, the total number of bumps given by the sample of inactives from each swarm was divided by the total number of bumps they received to yield a rate of propagation of bumping behavior in each swarm.

## Results

The population of wasps on the board rarely existed as one continuous cluster. Most of the time, from two to several dozen semi-distinct clusters were present, with a scattering of individuals on the outskirts of clusters and even fewer completely outside any cluster (Figure 1). After emigration began, clusters diminished in size as wasps became active and departed. After the breakup of a cluster of wasps, remaining individuals tended to either depart or join another cluster.

**Figure 3.**
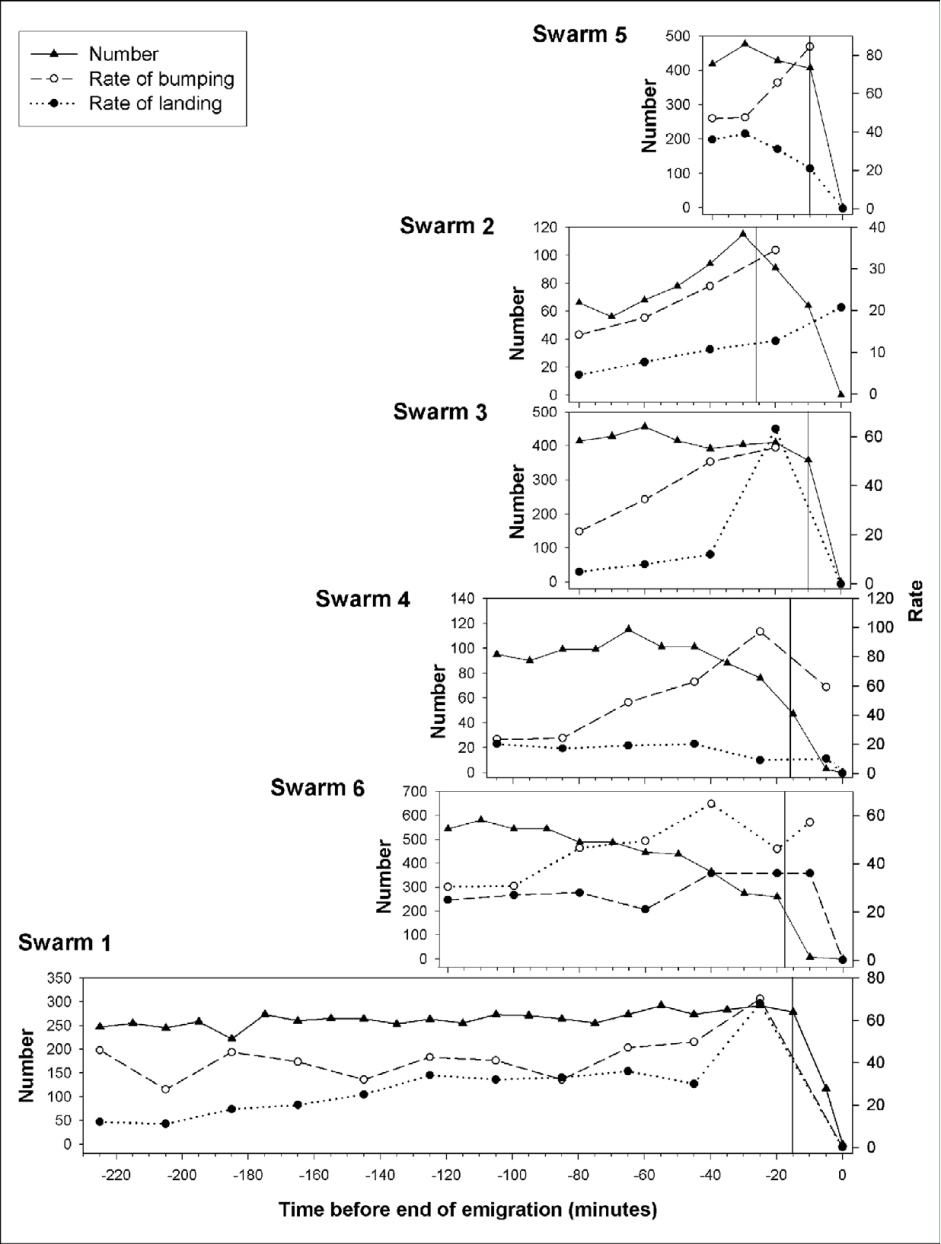
Behavior of *Polybia occidentals* scouts. Temporal patterns of scout activity up to and during the emigration of each swarm. The beginning of emigration in each swarm is represented by a vertical line. The end of emigration occurred at time = O. Number = number of wasps on the board. Rate of landing = number of scouts landing per 5-min period. Rate of bumping = mean number of bumps given by scouts per min. Note that y-axes are scaled differently.

The variation in observation time among swarms (Table 1) is due in part to the differing times required to coax the wasps from their twig onto the board, and also to the varying times required by swarms to locate a new nest site and emigrate to it. The maximum numbers of wasps on the boards at any one time represented 53–67% of the total swarm population, as determined by the predawn census (Table 1). After emigration began, 10–26 minutes passed before the last wasp left the board (Figure 3).

**Table 1.**
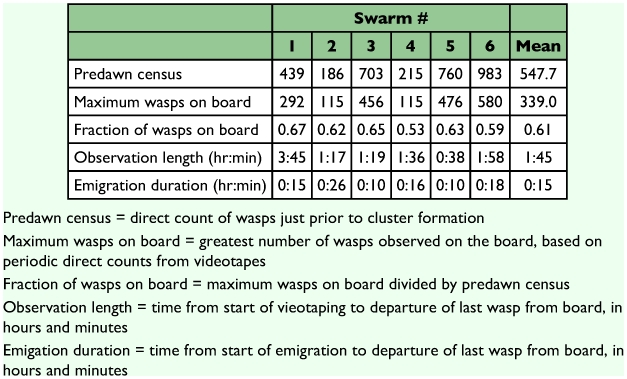
Data on *Polybia occidentalis* swarms used in the present study.

### Behavior of scouts

Because the camcorder was focused on the plane of the observation board, the videotapes revealed little about the flight paths of wasps as they arrived at and departed from the board. Hovering close to the clusters, which would have been visible on the tapes, was not observed. Once emigration began, visual observation of flying wasps in the greater vicinity of the board suggested that most of these were searching for the scent trail to the new nest site selected by the scouts.

Two patterns were observed with respect to scout behavior. First, the frequency of scout landings remained steady or increased slightly until after the emigration began, at which time landing frequencies decreased (Figure 3). An exception to this trend was swarm #2, in which scout landings continued to climb after the emigration began (Figure 3). The second pattern, observed in all six colonies, was that the rate of bumping by individual scouts steadily increased as emigration approached (Figure 3).

Since scouts were not individually marked, it is possible that on occasion the same scout was included more than once in our samples. However, because the high rates of landing by scouts (Figure 3) suggest that large numbers of scouts were active, we believe that the chance of repeat sampling of the same scout was small.

### Behavior of inactives

**Figure 4.**
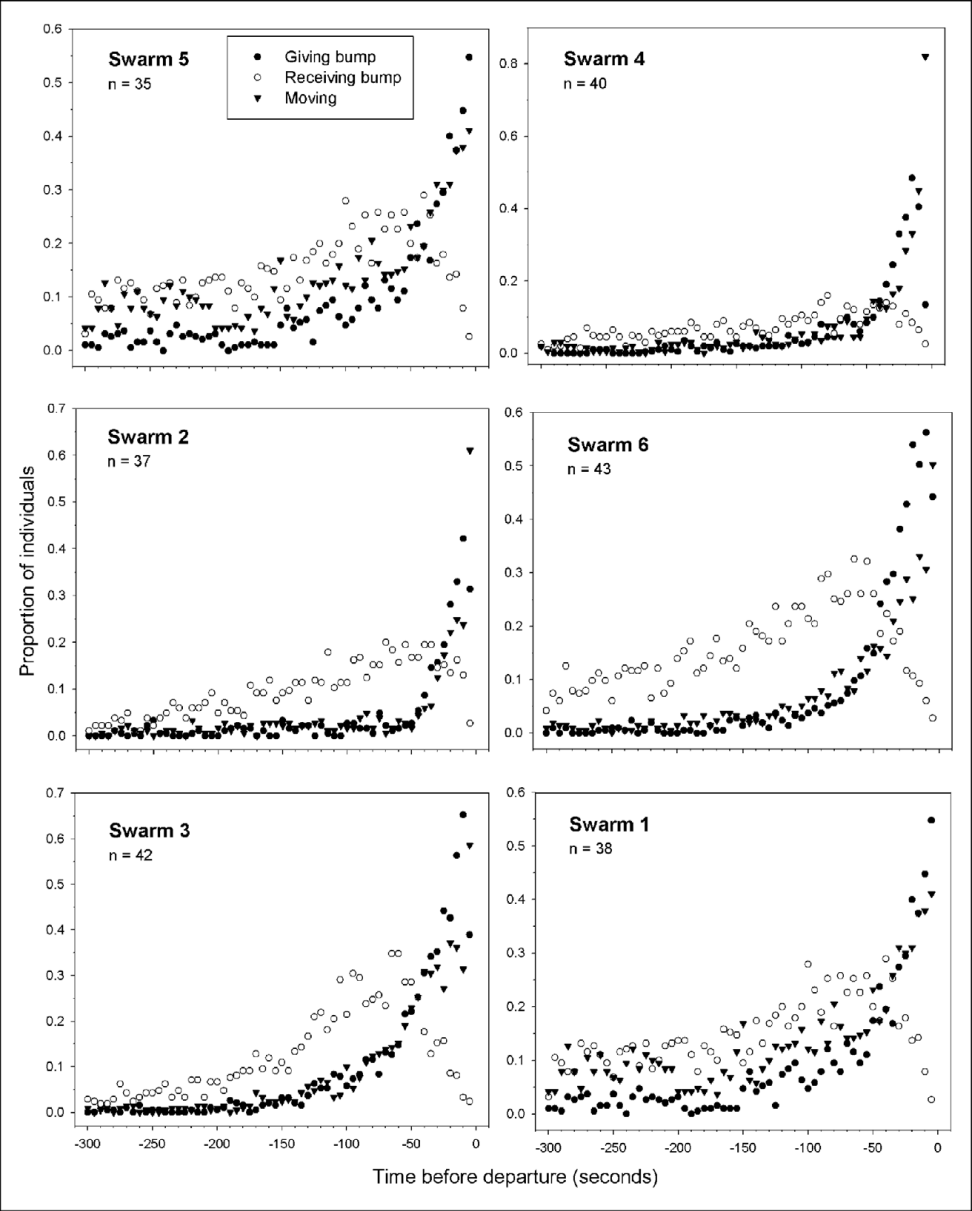
Pre-departure behavior of inactive *Polybia occidentals*. Proportions of inactive wasps engaged in three activities during the five minutes (300 sec) prior to the departure of each individual in the sample. When proportions do not total 1.0, as at the left-hand ends of the plots, the remaining proportions are made up of non-moving individuals. Individuals' absolute departure times are all different, but are aligned (at time = 0) so as to show sample-wide patterns. Note that y-axes are scaled differently.

Beginning five minutes before taking off, a typical inactive experienced a continually rising probability of being bumped by others (Figure 4). Approximately 100 seconds before takeoff the probability that the inactive was either moving or bumping others started to rise more steeply. At approximately 75 seconds before takeoff, the probability of receiving a bump reached its peak value before dropping off dramatically due to its replacement by increasing probabilities of moving and of bumping others (Figure 4). Trophallaxis events were rare (0.5 –5.4% of observations) and were not included in this analysis.

**Figure 5.**
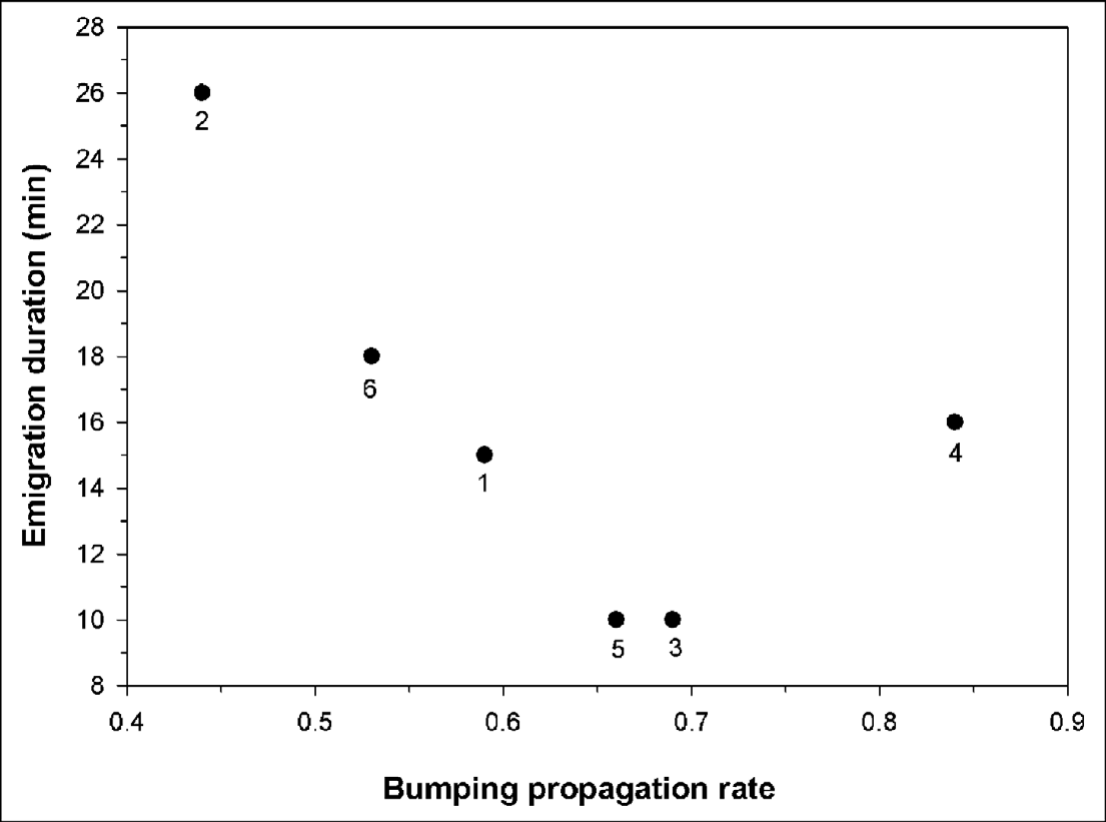
Time taken by swarms to emigrate as a function of bumping propagation rate. Rate of propagation = total number of bumps given divided by total received by the sample of inactives

The mean rate of propagation of bumping by inactives (total bumps given divided by total bumps received for each swarm–s sample of inactives) was 0.63 (range across swarms: 0.44–0.84) (Table 2). Although the duration of emigration appears to decrease with increasing propagation rate (Figure 5), the slope of the regression did not significantly differ from zero (p = .18). None of the patterns observed correlated with swarm size.

Viewing the videotape of the unmanipulated swarm on a twig did not reveal any behavior patterns in scouts or inactives that differed from those observed on the boards and reported here. In particular, in neither context were scouts seen burrowing their way through clusters or buzzing their wings as they moved over clustered wasps.

## Discussion

Since the maximum counts of wasps present on the board were 53–67% of the pre-dawn censuses and since the mortality rate for the census method used is very small (Bouwma et al. 2000), it was concluded that at any time at least 33–47% of the swarm was actively engaged in either foraging for nectar or scouting for new nest sites. This range is a minimum because the counts on the board included all foragers and scouts that happened to be present at the instant the count was taken.

The increase in bumping frequency by individual scouts as emigration approached may reflect increased enthusiasm for a site just inspected. Another possibility is that the strengthening of the pheromone trail leading to the new site stimulates scouts to bump at higher rates. While a similar trend is seen for scout landing frequencies in some cases, the relationship is not as consistent as the increase in bumping rates by scouts. This may be due to the possibility that while many scouts continue to land on the cluster once a site is chosen, others may be occupied with visiting the site for longer periods of time or reinforcing the pheromone trail, but still bump more frequently when they do visit the cluster. This would account for the lack of a dramatic rise in number of scout landings, but the maintenance of a high frequency of bumping during departure.

**Table 2.**
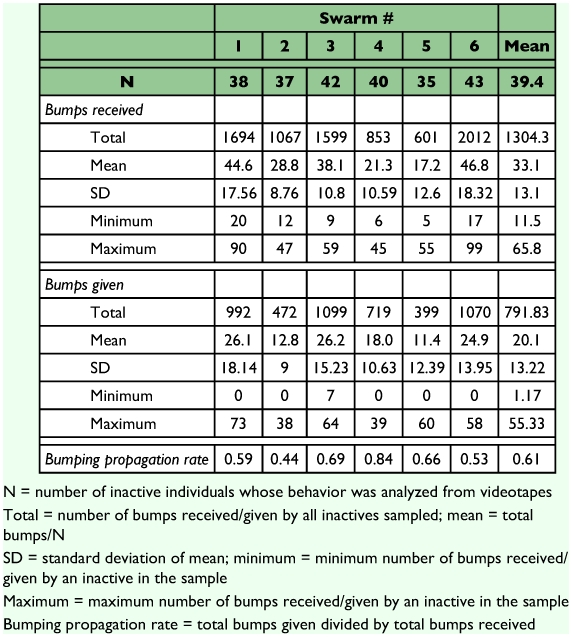
Behavior of sampled *Polybia occidentals* inactives.

The probability of an inactive–s receiving a bump peaked approximately 75 seconds before it took off. From this point on, the probabilities of receiving a bump, giving a bump, and moving total nearly 1.0, indicating that the typical individual has become fully activated. The most notable behavioral change observed in inactives, however, is that they go from being passive receivers of bumps to active producers of bumps before departing.

The short duration of emigration itself relative to how long scouts search for a new site indicates that emigration is a relatively brief and definable event in time. This in turn suggests that it is initiated by one or more cues or signals. Several observations of both scouts and inactives suggest that bumping is a signal that stimulates inactive individuals to become active and take off. First, individual scouts bump inactive swarm-mates at an increasing rate as the emigration event approaches, as expected if scouts use bumping as a signal to activate the rest of the swarm once a nest site has been selected. Second, activation and departure by an inactive are preceded by an increasing rate of being bumped. Again, a marked increase in the number of bumps received before becoming active would be expected if bumps were an activation stimulus. Third, after becoming active, previously inactive individuals tend to bump others before departing. That is, bumping is contagious, leading to its amplification through the swarm. Amplification further supports the notion that bumping is an emigration signal.

These results suggest the following scenario for the initiation of emigration of the temporary cluster. When a scout has visited a high-quality site, perhaps evaluating its attractiveness based on quorum sensing, as in honey bees (Seeley and Visscher 2004), she lays down a pheromone trail on foliage between the site and the cluster (Forsyth 1981; Jeanne 1981a, 1991). Upon returning to the cluster, the scout physically bumps into and runs over inactive individuals at a higher rate than if she had not found a favorable site. This may have the effect of stimulating other scouts on the cluster at the time to follow the trail. As more and more scouts visit the favorable site, the cluster will, as a result, receive much more bumping from the scout population. This increased rate of bumping causes the previously inactive individuals to become active, move, and propagate the signal by bumping others before departing to follow the pheromone trail to the new nest site. This hypothesis would be supported if activity among swarm members could be significantly increased experimentally by simulating bumping using models, or experimentally decreased by keeping scouts away from selected clusters on the swarm.

This scenario does not rule out the possibility that additional signals may be involved in this process. *Apoica pallens*, for example, apparently initiates emigration by releasing an airborne trail pheromone (Howard et al. 2002). In several species, including *P. occidentalis*, scouts or foragers have been observed to run rapidly, with wings buzzing, through groups of immobile wasps in clusters on the nest (Forsyth 1981). The effect is to break up these clusters, causing the inactive wasps to become active. Wing buzzing may be associated with pheromone release and dispersal. Pheromone release in other contexts on the nest is often accompanied by wing buzzing. An example is alarm-pheromone release in *P. occidentalis*, where the buzzing probably serves to disperse the chemical more quickly than by simple release (Jeanne 1981b). As stated above, no such wing buzzing was ever observed during the run-up to emigration in our *P. occidentalis* swarms, either on our observation boards or on the twig. It is possible that wing buzzing, with or without accompanying pheromone release, may be a stronger stimulus than mere bumping and is used in *P. occidentalis* when preparing for emigration directly from the nest, but not from the temporary cluster sites used in this study. In other genera, however, wing buzzing does occur on temporary swarm clusters away from the nest (Jeanne 1975; Naumann 1975) and is sometimes accompanied by rapid lateral shaking of the gaster (West-Eberhard 1982). We tentatively conclude that there is little evidence that pheromones were involved in the stimulation of flight in the swarms that were studied.

Other behavior may also be involved in inducing swarm movement. Following nest damage, foragers/scouts of *P. occidentalis* perform ‘loop flights,’ described as “relatively slow flights or circular motion with the wasp usually facing the nest and looping in a radius of 0.5 to 2 meters from the nest” (Forsyth 1978: 94). It has been suggested that these flights stimulate wasps in the cluster to become active and take flight (West-Eberhard 1982), but there is little direct evidence for this. The increase in loop flights in the minutes before emigration, as reported for *P. raui* (West-Eberhard 1982) may be an effect of other causes of flight, such as bumping, rather than its direct cause. Slow flights around the swarm as emigration got underway did occur in our swarms, but these were typically not close enough to clusters on the board to suggest that they could provide effective visual and/or chemical stimuli causing flight by inactives. Rather, it is likely that the slow, looping flights around the swarm are performed by non-scouts that have been activated to fly by bumping behavior and are now searching for emigration-trail scent marks left by the scouts. Nevertheless, the cause-effect relationships among the phenomena of bumping, wingbuzzing, and loop flights may differ among species and warrant more careful investigation.

Acceleration of the emigration process via the propagation of bumping by previously inactive wasps may be adaptive. Since wasps follow the emigration trail individually, the trail must be maintained long enough to ensure that all swarm members successfully reach the new site. Distributing some of the responsibility for stimulating inactives to depart onto the inactives themselves accelerates the process, thereby reducing the number of round trips the scouts must make to reinforce the pheromone trail leading to the new nest site. This in turn would reduce both the energy expended by scouts on flight and their risk of predation while en route. Further research will be necessary to determine if higher propagation rates translate into shorter emigration times.

Bumping behavior can be thought of as an activation signal, stimulating inactive individuals to become active and thus serving to regulate swarm emigration. Mechanical signals appear to regulate social activity in other contexts, as well. For example, biting behavior among workers of *P. occidentalis* stimulates foraging and thus plays a role in regulating task performance (O'Donnell 2001, 2006). Worker-on-worker vibration signals in *Apis mellifera* appear to have a similar effect (Hyland et al. 2007). Such signals may play a larger role in fine-tuning the regulation of social activities than has been recognized.
